# Report from the 2nd Cardiovascular Outcome Trial (CVOT) Summit of the Diabetes and Cardiovascular Disease (D&CVD) EASD Study Group

**DOI:** 10.1186/s12933-017-0508-8

**Published:** 2017-03-11

**Authors:** Oliver Schnell, Eberhard Standl, Doina Catrinoiu, Stefano Genovese, Nebojsa Lalic, Jan Skra, Paul Valensi, Dario Rahelic, Antonio Ceriello

**Affiliations:** 1 Forschergruppe Diabetes e.V., Munich, Ingolstaedter Landstrasse 1, 85764 Neuherberg, Germany; 2 Internal Medicine Department, Clinical County Emergency Hospital Constanta, Tomis Blvd, No. 145, 900591 Constanta, Romania; 3 Diabetes and Metabolic Disease Unit, IRCCS MultiMedica, Via Milanese 300, 20099 Sesto San Giovanni, MI Italy; 4 Clinic for Endocrinology, Diabetes and Metabolic Diseases, Clinical Center of Serbia, Faculty of Medicine, University of Belgrade, Dr Subotica 13, Belgrade, 11000 Serbia; 5 3rd Department of Internal Medicine, 1st Faculty of Medicine, Charles University, U Nemocnice 1, 128 08 Prague 2, Czech Republic; 6 Department of Endocrinology Diabetology Nutrition, CINFO, CRNH-IdF, Jean VERDIER Hospital, Paris 13 University, Avenue du 14 Juillet, 93140 Bondy, France; 7 Diabetes and Metabolic Disorders, Dubrava University Hospital, Avenija Gojka Šuška 6, 10000 Zagreb, Croatia

## Abstract

The 2nd Cardiovascular Outcome Trial (CVOT) Summit of the Diabetes and Cardiovascular Disease (D&CVD) EASD Study Group was held on the 20th–21st October 2016 in Munich. This second Summit was organized in light of recently published CVOTs on diabetes, with the aim of serving as a reference meeting for discussion on this topic. Along with presentations on the results of the most recently published CVOTs, panel discussions on trial implications for reimbursement and the perspective of cardiologists and/or nephrologists, as well as on CVOTs weaknesses and potentials constituted the heart of the program. Future activities of the D&CVD EASD Study Group in 2017 include an annual meeting in Milano and the 3rd CVOT Summit on Diabetes of the D&CVD EASD Study Group, in Munich (http://www.dcvd.org).

## Background

Since the 2008 FDA Guidance for industry “Diabetes Mellitus: Evaluating Cardiovascular Risk in New Antidiabetic Therapies in Type 2 Diabetes” [1] sponsors of all new antihyperglycemic drugs should demonstrate that the therapy will not result in an unacceptable increase in CV risk. Among the evaluated endpoints stand cardiovascular mortality, myocardial infarction and stroke, but also can include hospitalization for acute coronary syndrome, urgent revascularization procedures, and possibly other endpoints. Moreover, the FDA favors the enrollment of high-risk patients, such as those with relatively advanced disease, elderly patients, or under some degree of renal impairment. Along the lines of the FDA, the European Medicines Agency (EMA) [2] also requires an overall assessment of safety to exclude that a new drug increases the risk of macrovascular complications such as CVD.

## Main body

The 2nd CVOT Summit on Diabetes of the D&CVD EASD Study Group had the goal to present and discuss the results and implications from the most recently completed CVOTs (see summary Tables 1, 2).

**Table 1:** Overview of basic characteristics of CVOTs terminated in 2015 and published in 2016

	Study status	Drug	Drug class	Intervention	Primary outcome	N	Follow-up (years)	Start and estimated end date	Clinicaltrials.gov ID
EMPA-REG	Completed	Empagliflozin	SGLT-2 inhibitor	Empagliflozin 10 mg versus empagliflozin 25 mg versus placebo	CV death, MI, or stroke	7000	3.1	07.2010 to 04.2015	NCT01131676
LEADER	Completed	Liraglutide	GLP-1 inhibitor	Liraglutide versus placebo	CV death, MI, or stroke	9340	3.8	08.2010 to 12.2015	NCT01179048
SUSTAIN-6	Completed	Semaglutide	GLP-1 inhibitor	Semaglutide 0.5 mg versus semaglutide 1.0 mg versus placebo	CV death, MI, or stroke	3299	1.99	02.2013 to 01.2016	NCT01720446

**Table 2:** CVOTs terminated in 2015 and published in 2016: comparison of results versus placebo

Cardiovascular endpoints	EMPA-REG [4, 5]		LEADER [6]		SUSTAIN-6 [7]	
	Class	Hazard ratio (95% CI) *p* value	Class	Hazard ratio (95% CI) p-value	Class	Hazard ratio (95% CI) p-value
Primary composite MACE	CV death, MI, or stroke	0.86 (0.74–0.99) 0.04*	CV death, MI, or stroke	0.87 (0.78–0.97) 0.01	CV death, MI, or stroke	0.74 (0.58–0.95) <0.001/0.02*
Cardiovascular death	Primary end-point	0.62 (0.49–0.77) <0.001	Primary end-point	0.78 (0.66–0.93) 0.007	Primary end-point	0.98 (0.65–1.48) 0.92
Myocardial infarction	Primary end-point	0.87 (0.70–1.09) 0.23	Primary end-point	0.86 (0.73–1.00) 0.046	Primary end-point	0.74 (0.51–1.08) 0.12
Stroke	Primary end-point	1.18 (0.89–1.56) 0.26	Primary end-point	0.86 (0.71–1.06) 0.16	Primary end-point	0.61 (0.38–0.99) 0.04
Hospitalization for unstable angina	Secondary end-point	0.99 (0.74–1.34) 0.97	Extended Primary end-point	0.98 (0.76–1.26) 0.87	Extended Primary end-point	0.82 (0.47–1.44) 0.49
Hospitalization for heart failure	Secondary end-point	0.65 (0.50–0.85) 0.002	Extended Primary end-point	0.87 (0.73–1.05) 0.14	Extended Primary end-point	1.11 (0.77–1.61) 0.57
Primary composite MACE	Event rate (%) active group		Event rate (%) active group		Event rate (%) active group	
	10.5		13.0		6.6	

Non-cardiovascular endpoints	No. (%) p value	No. (%) p value	No. (%) p value
Renal event	5.2	5.7	3.8
Acute pancreatitis	0.3^$^	0.4 0.44	0.54
Hypoglycemia events	1.3	3.3 0.02	22.4^₭^

* Superiority test; ^$^ average across all age ranges; ^₭^ Severe hypoglycemia as defined by ADA

Key topics and aims of the 2nd CVOT Summit on Diabetes were:

1. Discuss on implications of SGLT-2 inhibitors and CVOT results on renal outcomes.
  Glycemic control was highlighted as a key strategy for renal protection since it reduces the risk of albuminuria and dialysis. By preventing hyperfiltration, SGLT-2 inhibitors like empagliflozin reduce intraglomerular pressure, and in addition to standard care, can reduce the risk of progression of CKD. Other potential explanations for the beneficial cardiovascular and renal effects of SGLT-2 inhibitors might be by improving oxygen delivery and/or providing hydroxybutyrate as heart fuel, but potentially also through an increase in glucagon. In summary, SGLT-2 inhibitors have the potential to be as revolutionary a therapy as RAAS inhibition.
2. Summarize the key learnings from recent CVOTs (namely, EMPA-REG, LEADER & SUSTAIN-6), especially with respect to heart failure (HF).
  Diabetes markedly increases the risk for HF even at a young age. Furthermore, HF in diabetes has a poor prognosis, with up to 10 times increased mortality in comparison with patients with diabetes but without HF. Even though strict glycemic control has not shown a reduction of HF events, results of recent CVOTs have shown that certain antihyperglycemic therapy can, independently of glycemic control, lead to a decrease of HF risk. However, evidence for high rates of undiagnosed HF in recent CVOTs calls for further attention into HF characterization in the context of CVOTs and/or antidiabetic treatments [3]. On the other hand, the analysis of the results on HF observed for these latest trials call for further research into the possible biological mechanisms (such as the role of cardiac fuel overload (lipo-gluco-toxicity) vs renal glucotoxicity; or the potential association with hypoglycemia) leading to them.
3. Present an update on lipid studies, and reflect on their influence on diabetes and CVOT design and results.
  On the topic of lipid therapy in the context of diabetes, apart from the need for a risk-based LDL-cholesterol/non-LDL-cholesterol goal determination, treatment recommendations include statins as first line approach, to be possibly complemented with ezetimibe or PCSK9 inhibitors, fibrates/omega-3 fatty acids in selected patients. Furthermore, to combat hypertriglyceridemia, life style modification/glucose control are highly recommendable.
4. Include the perspectives of other health professionals like cardiologists in the discussion of future implications of CVOTs.
  One of the general points of agreement between diabetologists and cardiologists was the importance of improving on study design. Issues related to endpoint selection and under-powering, short study duration and lack of head-to-head comparisons were highlighted. Reconsideration of end-points, study population selection criteria, comparator selection and statistical analysis were among the suggestions derived from the discussion to improve on CVOT results.
5. Promote discussion on the results and implications of CVOTs for therapy and reimbursement.
  Despite the potential of CVOT results to affect treatment guidelines and reimbursement plans in Europe, so far in Germany, for instance, CVOT results have not affected much drug pricing. Partly due to the different “standard of care according to local guidelines”, which may lead to insufficient glycemic control (and antihypertensive treatment). But also to the insufficiently individually defined treatment escalation and treatment goals pre-randomization and the lack of comprehensive analyses on regional influences, which hamper the use of CVOT results as regulatory evidence.
6. Enforce cross-sectorial communication among the scientific community, trial sponsors and regulatory and reimbursement authorities.

Other questions debated during the 2nd CVOT Summit on Diabetes were the following:

1. What are the key learnings from CVOTs in 2016?
  One of the main points of discussion. In general it was agreed that the latest CVOTs have shown that glucose lowering drugs can decrease CV morbidity and mortality. However, the exact mechanisms involved are still unknown. To which extent is it due to glucose-lowering mechanisms or by means of non-glycemic effects like weight loss or blood pressure control, remains under question. The positive cardiovascular effects observed in this year’s published trials on GLP-1 RA, LEADER and SUSTAIN-6, demonstrated clear within-class differences, especially when compared to results observed in trials like ELIXA. This variability of results observed for drugs from the same class (GLP-1 RA) raises questions as to whether it is reasonable to expect a class effect for anti-hyperglycemic drugs. Moreover, the dissimilarities between trials with respect to single cardiovascular endpoints raises the point of mechanistic differences between GLP-1 RA and SGLT-2 inhibitors. While effects driven by GLP-1 RA would be mainly mediated by endothelial changes leading to improved myocardial perfusion, SGLT-2 inhibitors would exert their action by mediating hemodynamic changes.
  For all the above, it is necessary therefore to deepen the knowledge on the mechanism of action of these drugs, and to that effect initiate new trials aiming on that direction.
2. Can CVOTs lead to changes in treatment algorithms?
  The general answer to this question was affirmative under a certain set of conditions, namely that results are consistent among trials with the same drug and therefore there is an independent confirmation of results. Furthermore, trial design must be of such quality that allows results validation (pre-specified statistical analysis plan, P < 0.01 for primary endpoint and consistent results in major subgroups…). Finally, drugs examined by CVOTs must show a certain strength of demonstrated benefits and/or safety signals to be considered in the guideline recommendations by professional associations.
3. Can CVOT results be extrapolated to broader populations?
  In general, it seems reasonable to assume CV safety in broader populations given the high risk profile of the studied groups, even when this very same condition, the inclusion of high risk populations in CVOT design, might be a limiting factor for extrapolation. However, result extrapolation might be possible to some extent provided that specific eligibility criteria of target populations are satisfied and that subgroup analysis of CVOT subjects show consistency of results.

## Conclusion

 The 2nd Meeting on CVOTs in Diabetes of the D&CVD EASD Study Group was a successful scientific meeting where results from the most recently completed trials were discussed in a cross-functional international setting. The Summit discussed on the learnings and limitations of current CVOT study design. Their impact on treatment guidelines and reimbursement and viewed CVOTs results needs to be considered under the specialized perspectives of the nephrologist, cardiologist and diabetologist.

Scientific activities of the D&CVD EASD Study Group in 2017 include the annual meeting in Milano and the 3rd CVOT Summit on Diabetes of the D&CVD EASD Study Group, in Munich (http://www.dcvd.org).

## Abbreviations

CKD: chronic kidney disease
CVD: cardiovascular disease
CVOT: Cardiovascular Outcome Trial
D&CVD: Diabetes and Cardiovascular Disease
EASD: European Association for the Study of Diabetes
EMA: European Medicine Agency
FDA: Food and Drug Administration
GLP-1: glucagon-like-peptide 1
HF: heart failure
MI: myocardial infarct
SGLT: 2-sodium glucose linked transporter 2
