# Correction to: Report from the 2nd Cardiovascular Outcome Trial (CVOT) Summit of the Diabetes and Cardiovascular Disease (D&CVD) EASD Study Group

**DOI:** 10.1186/s12933-017-0616-5

**Published:** 2017-10-18

**Authors:** Oliver Schnell, Eberhard Standl, Doina Catrinoiu, Stefano Genovese, Nebojsa Lalic, Jan Skra, Paul Valensi, Dario Rahelic, Antonio Ceriello

**Affiliations:** 1 Forschergruppe Diabetes e.V., Munich, Ingolstaedter Landstrasse 1, 85764 Neuherberg, Germany; 2 Internal Medicine Department, Clinical County Emergency Hospital Constanta, Tomis Blvd, No. 145, 900591 Constanta, Romania; 3 Diabetes and Metabolic Disease Unit, IRCCS MultiMedica, Via Milanese 300, 20099 Sesto San Giovanni, MI Italy; 4 Clinic for Endocrinology, Diabetes and Metabolic Diseases, Clinical Center of Serbia, Faculty of Medicine, University of Belgrade, Dr Subotica 13, Belgrade, 11000 Serbia; 5 3rd Department of Internal Medicine, 1st Faculty of Medicine, Charles University, U Nemocnice 1, 128 08 Prague 2, Czech Republic; 6 Department of Endocrinology Diabetology Nutrition, CINFO, CRNH-IdF, Jean VERDIER Hospital, Paris 13 University, Avenue du 14 Juillet, 93140 Bondy, France; 7 Diabetes and Metabolic Disorders, Dubrava University Hospital, Avenija Gojka Šuška 6, 10000 Zagreb, Croatia; 8 Institut d’Investigacions Biomèdiques August Pi i Sunyer (IDIBAPS) and Centro de Investigacion Biomèdica en Red de Diabetes y Enfermedades Metabolicas Asociadas (CIBERDEM), Barcelona, Spain; 9 Department of Cardiovascular and Metabolic Diseases, IRCCS Multimedica, Sesto San Giovanni, Milan, Italy

## Correction to: Cardiovasc Diabetol (2017) 16:35 DOI 10.1186/s12933-017-0508-8

Following publication of the original article [1], author Antonio Ceriello requested that a correction be published in relation to his affiliations. His correct affiliations have been updated in this erratum. This correction is very important for the correct assignment of funds to his Institutions.
